# Modeling Podocyte Ontogeny and Podocytopathies with the Zebrafish

**DOI:** 10.3390/jdb11010009

**Published:** 2023-02-20

**Authors:** Bridgette E. Drummond, Wesley S. Ercanbrack, Rebecca A. Wingert

**Affiliations:** Department of Biological Sciences, Center for Stem Cells and Regenerative Medicine, Center for Zebrafish Research, Boler-Parseghian Center for Rare and Neglected Diseases, Warren Center for Drug Discovery, University of Notre Dame, Notre Dame, IN 46556, USA

**Keywords:** podocyte, nephron, development, zebrafish, retinoic acid, notch, *brca2*, *osr1*, *wnt2ba*

## Abstract

Podocytes are exquisitely fashioned kidney cells that serve an essential role in the process of blood filtration. Congenital malformation or damage to podocytes has dire consequences and initiates a cascade of pathological changes leading to renal disease states known as podocytopathies. In addition, animal models have been integral to discovering the molecular pathways that direct the development of podocytes. In this review, we explore how researchers have used the zebrafish to illuminate new insights about the processes of podocyte ontogeny, model podocytopathies, and create opportunities to discover future therapies.

## 1. Introduction

### 1.1. Overview of Kidney Organization and Function

**Figure 1:**
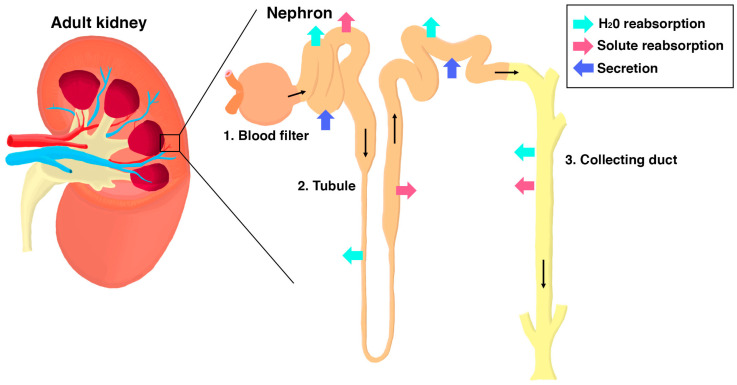
The nephron is the functional subunit of the kidney. (**left**) Schematic of a representative adult (metanephric) human kidney which contains millions of nephrons. (**right**) Each nephron has three major components: (1) the blood filter, (2) the tubule, (3) the collecting duct. Blood flows into the filter where a filtrate is formed. The filtrate then enters into the tubule where water and/or solutes are selectively reabsorbed or secreted. Regional differences in tubule epithelial cell characteristics establish a defined sequence of modifications. From the tubule, the waste product passes through the collecting duct where final adjustments are made in water and electrolytes before the urine is passed out of the kidney. Some example physiological functions (arrows) are indicated.

**Figure 2:**
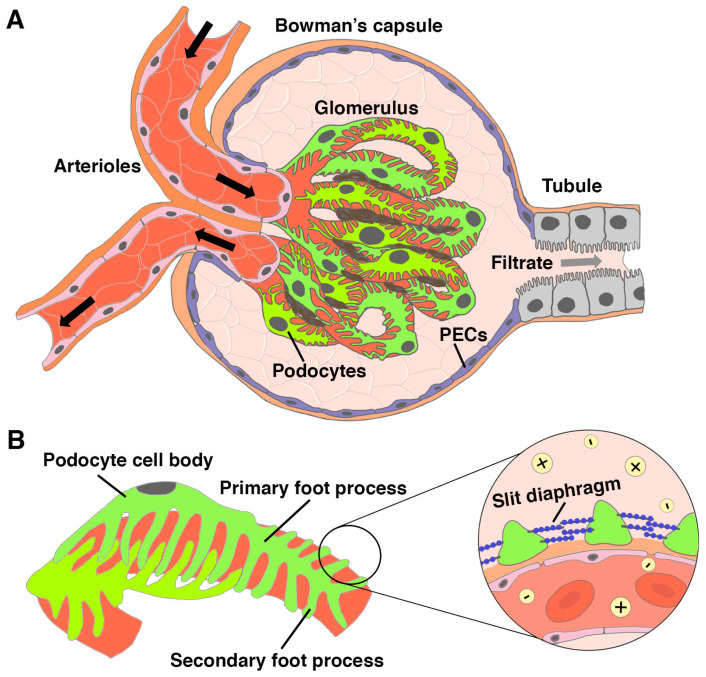
Blood filtration is made possible by podocytes. (**A**) The Bowman’s capsule connects to the nephron tubule and completely surrounds the glomerulus, a ball of capillaries that receive blood flow from arterioles. Specialized epithelial cells known as podocytes wrap around the glomerulus, while the parietal epithelial cells (PECs) line the Bowman’s capsule. Mesangial cells (brown color) lie between and around the capillaries, creating a supporting structure for the tuft. (**B**) Podocytes have a series of cellular extensions known as primary and secondary foot processes. A magnified cross section of podocytes reveals the slit diaphragm, which consists of protein:protein cross bridges that connect adjacent foot processes. The slit diaphragm only allows for water and small, charged particles to enter the Bowman’s space and proceed to the nephron tubule.

The kidney is an integral organ that performs key physiological tasks through the work of its individual functional units known as nephrons (Figure 1) [1]. The first essential job of each nephron is blood filtration. This process occurs in the renal corpuscle, which is where the vascular system interfaces directly with the start of the nephron [2]. The blood flows into the renal corpuscle through arterioles, where it passes through a dense tuft of capillaries called the glomerulus (Figure 2A). The capillary tuft is interspersed with mesangial cells that provide a support structure and influence filtration pressure at the glomerulus [2]. If the glomerulus is a baseball, the mitt that holds it is the Bowman’s capsule, which is an enclosure lined with parietal epithelial cells (PECs) (Figure 2A). A crucial epithelial cell type within the renal corpuscle are podocytes, which adhere to one side of the specialized glomerular basement membrane (GBM), in opposition to the fenestrated endothelial cells that comprise the capillary walls on the other side [2,3].

Podocytes are often likened to an octopus, as they have long, tentacle-like cellular extensions known as foot processes (Figure 2B) [4]. Electron microscopy studies have revealed that podocytes contain hundreds to thousands of primary and secondary foot processes that fit together such as intricate puzzle pieces along each capillary of the glomerulus [5,6,7]. Neighboring podocytes are physically connected to each other by a network of protein extensions that project from the individual foot processes, creating a sieve-like barrier termed the slit diaphragm (Figure 2B) [8]. The GBM has sieve-like properties as well due to its composition of collagen fibers and other proteins and also repels negatively charged plasma proteins and negatively charged glycoproteins [8]. Together, the combination of fenestrated capillary membrane, GBM, and podocytes with their slit diaphragm comprise the glomerular filtration barrier (GFB). The GFB is a highly specialized interface which allows passage of smaller particles such as water, sugars, and salts into the Bowman’s space, while circulating blood cells and bulky molecules such as large proteins are retained within the glomerulus [9,10]. The materials that successfully pass through the GBM and slit diaphragm form a filtrate that proceeds into the nephron tubule.

As the filtrate passes through the tubule, materials are selectively absorbed and secreted by specialized epithelial cells that occupy discrete regions, termed “segments” [1,11]. For example, the proximal segments perform the bulk of reabsorption, especially of organic molecules, while the distal segments fine-tune the amount of salts and water within the filtrate [11,12,13,14,15,16,17,18,19]. The nephron tubule is attached to the collecting duct, which drains the concentrated filtrate for excretion into the ureters and bladder [1]. The two kidneys in our body, each composed of 300,000 to 1.8 million nephrons, filter all of the blood in our body almost 30 times daily to produce 1–2 quarts of urine [20]. As indispensable and multifaceted as this system is, it is no surprise that dysregulation of the renal system results in an assortment of diseases.

### 1.2. Podocytes in Kidney Injury and Disease States

In the event of injury, the mammalian nephron has limited potential for self-repair [21,22,23]. As the front line of defense for the nephron, podocytes are the most susceptible to injury and death, and the delicate foot processes and extensions are often the first to be damaged upon injury (Figure 2) [24,25,26]. Alterations to the podocytes disrupt the fine filtering net of the slit diaphragm and can lead to podocyte effacement, where morphological changes flatten and gradually retract their foot processes, or even the loss of podocytes due to detachment from the GBM. Podocyte alterations compromise size-selective filtration, leading to proteinuria, in which the tubule will be bombarded with proteins, other large molecules, and even vascular cells [27,28]. Over time, this leads to tubular cell damage and ultimately to the atrophy of the nephron, along with other pathologies such as renal fibrosis, as further discussed in the following paragraphs. If these events are replicated in enough regions of the kidney(s), renal function can cease to support the metabolic demands of the body and require replacement therapy.

Further complicating matters is the fact that podocytes are thought to be post-mitotic after birth in humans, and no evidence has been uncovered yet suggesting that podocytes are capable of regenerating in humans [29,30]. In keeping with this fact, a multitude of kidney diseases originate in the glomerulus with the initiating insult of podocyte injury and attrition [30,31,32,33,34,35]. After a nephron dies, fibrosis occurs in the interstitial spaces between nephrons. This renal fibrosis can cause a chain reaction of additional nephron stress and death, which eventually decreases organ functionality [30,31,32,33,34,35]. This is the case in acute kidney injury (AKI), chronic kidney disease (CKD), and end stage renal disease (ESRD), as the kidney suffers an initial injury, advanced fibrosis, and total loss of function, respectively [36,37,38,39,40,41,42]. When kidney function is reduced by more than 85%, this signifies that CKD has progressed to end stage renal disease (ESRD) or kidney failure [42].

CKDs can be caused by environmental stressors such as toxicity or trauma [41,42]. Blood with an abnormal composition that enters the glomerulus can potentially damage the podocytes. If a patient has underlying metabolic, immune, or blood circulation issues, this can be detrimental to podocytes [31]. Equally detrimental are exposures to infectious pathogens and toxins. Physical force aimed at the trunk also risks damaging a kidney. Further, other major factors such as aging and genetic background can be leading culprits in the decline of renal function [43]. Several other diseases are causative of CKD or related to CKD in some way. For example, between 30–40% of diabetes patients exhibit CKD, though the full reasons behind this remain unclear [41,42]. Other examples are cardiovascular disease, hypertension, anemia, and lupus [41,42]. Between 7 and 12% of people worldwide are currently affected by some stage of CKD [41,42]. We are currently experiencing a CKD/ESRD epidemic greater than any other era in history, with a doubling of patients on dialysis within the last 20 years alone [42,43]. To combat this crisis, there are several therapeutic options such as angiotensin blockers, sodium-glucose co-transporter-2 (SGLT-2) inhibitors, G-protein-coupled receptor blockade, and endothelin or EP receptor blockade, as well as other candidates that may potentially be used [44].

Congenital anomalies of the kidney and urinary tract (CAKUT) are a global issue that can manifest as dozens of phenotypes [45,46,47,48,49], many of which lead to CKD or, in the most severe cases, premature death. During embryonic development, a kidney may be malformed, such as in cases of the horseshoe kidney, or have a smaller overall size with fewer nephrons, known as renal hypoplasia [45,46,47,48,49]. In the most severe cases of kidney development gone wrong, one or more kidneys fail to form, known as unilateral and bilateral agenesis, respectively [45,46,47,48,49]. An estimated 20–30% of congenital malformations involve kidney issues, and one reason for this is that 50% of CAKUT cases occur as part of a multi-organ condition, such as the syndrome known as vertebral defects, anal atresia, cardiac defects, tracheal-esophageal abnormalities, renal and radial anomalies, and limb abnormalities (VACTERL) [50]. The embryonic diagnosis of CAKUT has improved at a similar pace to the advancement of ultrasound technology. However, understanding the genetic landscape of kidney development may reveal new aspects and possible therapeutic interventions.

Due to the lack of treatment options for kidney diseases, in recent years researchers have made seminal efforts to grow patient-specific kidney organoids for drug testing and transplantation [51,52,53,54,55,56,57,58,59,60,61]. While numerous cell types of the kidney are present in these organoids, including podocytes, there have been many ongoing efforts to elaborate on these early methods and to identify the additional genetic factors that might be needed to create fully differentiated and functional kidney cells [62,63]. Ongoing studies of podocyte development and injury in animal models are continuing to provide new insights relevant to understanding the basis of renal diseases and the formulation of new therapies. In the next section of this review, we will provide an overview of nephrogenesis with a focus on the emergence of the podocyte lineage. In the subsequent sections of this review, we discuss how the zebrafish has been utilized to delineate the mechanisms of podocyte development and model podocytopathies, and we explore future directions for the field.

## 2. Emergence of Podocytes during Kidney Organogenesis

**Figure 3:**
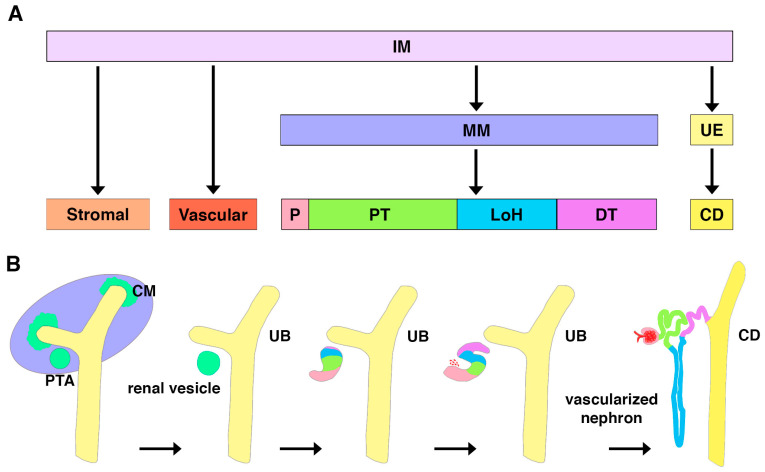
Mammalian mesodermal and nephron development. Simplified lineages and anatomical stages of the developing mammalian kidney are depicted. (**A**) The intermediate mesoderm (IM) will form the stromal, vascular, metanephric mesenchyme (MM), and ureteric epithelium (UE). MM develops into the podocyte (P), proximal tubule (PT), Loop of Henle (LoH), and distal tubule (DT), while the UE results in the collecting duct (CD). (**B**) Anatomical stages are shown first with condensation resulting in cap mesenchyme (CM) and pretubular aggregates (PTA) around the ureteric bud (UB). CM and PTA are followed by formation of the renal vesicle, comma-shaped body, S-shaped body, and vascularized nephron. Adapted from [60].

In vertebrate embryos, three tissue types become apparent: endoderm, mesoderm, and ectoderm. Fate mapping technology has revealed how the various subpopulations and mixtures of the germ layers generate organs. In amniotes, the genitourinary system arises from the intermediate mesoderm (IM) (Figure 3A) [64]. The earliest form of the kidney is the pronephros. While functional in lower vertebrates such as fish, the pronephros is vestigial in mammals [65,66]. This structure degenerates to give rise to the mesonephros, which is the final kidney form in fish and amphibians, but in mammals it is followed by the metanephros [65,66]. In mammals, the mesonephros provides transitory renal function during gestation while the terminal kidney, or metanephros, is developed [67].

Across vertebrates, each kidney form is composed of nephrons, but they have increasingly complex architectural arrangements [22,68,69,70]. For example, the genesis of the complex metanephros utilizes branching morphogenesis with intricate reciprocal interactions to form thousands of nephrons arranged around a central collecting duct system. At approximately 5 weeks of gestation, metanephros formation begins in human embryos with the outgrowth of the future collecting duct, known as the ureteric bud [64,65,66,67,68]. The ureteric bud invades the metanephric mesenchyme, which is composed of aggregates of multipotent cells expressing transcription factors such as Lim homeobox 1 (LHX1) and paired box gene 2 (PAX2) [64,65,66,67,68]. Reiterative branching morphogenesis of the ureteric bud will give rise to a ramified collecting duct system, and cross-talk between this ductal network and the surrounding mesenchyme induces nephrogenesis [64,65,66,67,68]. Nephrons are triggered to form by signals that cause groups of mesenchyme cells to undergo an epithelial transition to form a renal vesicle [64,65,66,67,68]. Differentiation cues lead to the renal vesicle expanding and organizing into the comma shaped body stage and then the S-shaped body stage until the mature, segmented nephron merges and fuses to the collecting duct (Figure 3B) [64,65,66,67,68]. The processes of renal vesicle introduction, patterning, growth, and morphogenesis repeat hundreds, thousands, or even millions of times in mammals. In human embryos, for example, nephrons begin to form at 9 weeks, and no new nephrons are formed after 36 weeks, or approximately 4 weeks before birth [67,71,72]. Some mammals, such as the mouse, exhibit a protracted period of nephrogenesis immediately following birth, but after this unique post-natal period there is no subsequent nephrogenesis [64,65,66]. This is surprising because many organs, such as the brain, continue to develop in infants [71]. Interestingly, in teleosts such as the zebrafish, the terminal kidney continues to grow throughout their lifetime and undergoes neonephrogenesis upon injury—processes that both include the generation of new podocytes and will be discussed later in this review [73,74,75,76].

Single-cell analysis has revealed that one of the first fate choices that a mammalian nephron progenitor makes during development is whether or not it will become a podocyte or proximal tubular cell [77,78]. Podocyte precursors can first be seen in a segment of cells in the S-shaped phase of development, which is marked by Wilms tumor 1 (WT1) [79]. During the S-shaped phase, podocyte precursors appear columnar in shape and are connected by tight junctions [7]. In the capillary loop stage, developing vasculature continues to enfold into podocyte and GBM epithelia, and podocytes begin to sprout primitive foot processes [7,24,31,79]. As the podocytes continue to mature, the distances between the cell bodies widen as more and more foot processes and secondary extensions from foot processes protrude [7,79]. The foot processes of different podocytes interlink with each other before becoming attached to the GBM [79,80,81,82]. Once podocytes have matured, the initial tight junction connections are replaced by the slit diaphragm, and foot processes and extensions appear largely uniform from podocyte to podocyte [7,79]. At each stage in the process of podocyte development, complex genetic regulatory networks are at play to advance the cell towards maturation [79,80,81,82]. In order to elucidate these networks, a variety of animal models have been utilized due to the high degree of conservation of podocyte features across vertebrate kidney forms [83].

## 3. Zebrafish as a Model for Podocyte Development

*Danio rerio*, commonly known as the zebrafish due to their blue and silver striped pigmentation patterns, are small, tropical freshwater fish that have been used for biological research studies since the 1960s [84,85,86,87]. The zebrafish genome is conserved with that of higher vertebrates, including humans [88]. This genetic similarity, along with a number of other attributes, has led to the use of this species as a preeminent model for disease modeling, drug screening, and developmental genetics [84,85,86,87]. Zebrafish exhibit broadcast spawning and can produce 100–1000 embryos per week, which is conducive to experimental approaches such as high-throughput screening [84,85,86,87]. Zebrafish embryos are very amenable to study as they develop *ex utero*, are transparent during early stages, and exhibit rapid organogenesis in the first 5 days of life [89]. Robust methodologies have been formulated for cellular and molecular studies [90,91]. The zebrafish embryonic kidney, or pronephros, was examined during early genetic screens and found to display a simple architecture of two nephrons that shared a single renal corpuscle, thereby offering a new opportunity to examine nephrogenesis [92], as well as nephron cell types similar to mammals [93,94]. Since this time, the zebrafish has emerged as an attractive model for studying kidney ontogeny, physiological, and renal disease [95,96,97,98,99].

**Figure 4:**
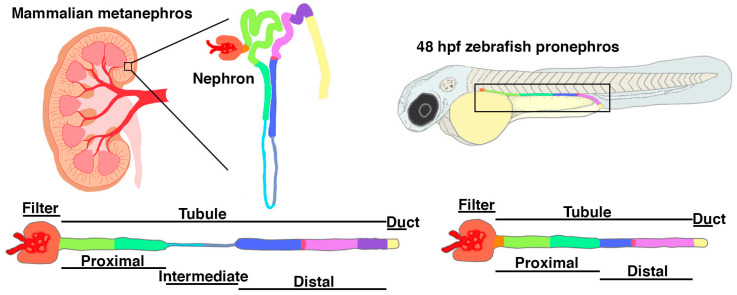
Nephron composition and segment pattern is conserved between zebrafish and mammals. Nephrons in the mature mammalian metanephric kidney are composed of a renal corpuscle followed by proximal, intermediate, and distal tubule segments. This anatomical pattern is conserved in the zebrafish pronephros, which form a renal corpuscle at the midline which sends filtrate to the pair of nephrons that contain a series of proximal and distal segments. The podocytes and tubule cells in zebrafish express a shared suite of conserved genetic markers in common with mammals.

As discussed briefly in the previous section, the earliest and simplest form of any vertebrate kidney is the pronephros. The zebrafish pronephros is made up of two nephrons, which are initially linear and situated in a parallel arrangement on either side of the embryonic trunk, making them highly accessible for visualization during development [92]. Further, this simple architecture enables the precise visualization of each nephron lineage as it emerges [92,93,94]. The nephrons arise from mesenchymal renal progenitors that undergo an epithelial transition during the first day of development [100,101], during which time they make lineage decisions based on the genetic cascades that are increasingly being delineated [102,103,104,105,106,107,108,109,110,111,112,113,114,115,116,117,118]. In addition to their favorable anatomical position within the embryo, the nephrons also exhibit genetic and morphological similarities in kidney organogenesis to mammals. For example, *LHX1* and *PAX2* are orthologous to *LIM homeobox 1a* (*lhx1a*) and *paired box 2a* (*pax2a*), which are also demonstrated renal progenitor markers in zebrafish [104,119]. Additionally, zebrafish podocytes rapidly develop and morphologically resemble mammalian podocytes as seen with orthologous markers such as *wilms tumor 1a, wilms tumor 1b, nephrosis 1, congenital Finnish type (nephrin), nephrosis 2, idiopathic, steroid-resistant (podocin)* (*wt1a/b*, *nphs1*, and *nphs2*) and *WT1, NPHS1, NPHS2*, respectively [120,121,122,123,124,125,126,127]. The zebrafish nephron also has conserved solute transporter genes that are expressed in proximal and distal segments that are analogous to those comprising the mammalian nephron (Figure 4) [93].

The zebrafish pronephros begins to filter the embryonic circulation at approximately 48 h post fertilization (hpf), although the glomerulus undergoes further maturation and becomes more size-selective by 4 days post fertilization (dpf) [128]. This rapid formation of the renal corpuscle provides an unprecedented opportunity to determine the underlying mechanisms of this process in vivo and in real time, as demonstrated by seminal studies (e.g., [92,128]). The corpuscle of Stannius (CS) and the interrenal gland are endocrine glands that are associated with the pronephros and develop concomitantly [93,106,112,129,130,131,132,133,134,135,136]. The CS regulates calcium in fish, while the interrenal gland is the teleostean equivalent of the mammalian adrenal gland and produces steroid hormones [106,112,129,130,131,132,133,134,135,136]. Among the greatest advantages of the zebrafish model is the overall simplicity of the nephron structure, which allows for ease of visualization after experimental manipulation, which we discuss in the following section.

### 3.1. Visualization of Podocyte Development Using Zebrafish

**Figure 5:**
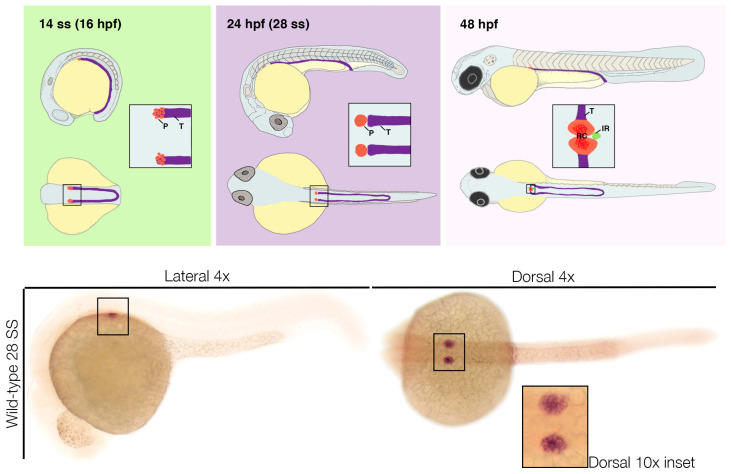
Podocyte development in the zebrafish pronephros. (**top, left**) Podocytes (P) arise from a rostral subset of the renal progenitors, adjacent to the tubule (T) progenitors. (**top, middle**) At 24 hpf, podocyte precursors can be seen as two bilateral clusters of cells, while the two nephron tubules are positioned along the length of the trunk, just dorsal to the yolk sac extension. (**top, right**) By the 48 hpf stage, the podocyte precursors have migrated to the midline, fused, and become associated with vasculature. This event allows for the renal corpuscle (RC) to filter blood and for the pronephros to function. The interrenal gland (IR), an endocrine gland associated with podocytes, has also fused at the midline by this point. (**bottom**) Zebrafish embryo stained using the procedure of whole mount in situ hybridization to visualize the podocytes based on their specific expression of the transcription factor *wt1b*. Boxed areas in lateral and dorsal views indicate the location of the podocytes.

One standard methodology to visualize podocytes, as well as other components of the pronephros and its associated organs, in fixed samples is through whole mount in situ and fluorescent in situ hybridization [137,138,139,140,141,142,143,144]. Podocytes arise from the anterior most group of renal progenitor cells, which appears as two stripes of cells situated laterally to endothelium/hematopoietic precursors in the lateral plate mesoderm of the young zebrafish embryo (Figure 5). Early markers of zebrafish renal progenitors include *pax2a, pax8, lhx2a*, and *hnf1ba/b* [92,93,104,145,146,147]. The earliest possible appearance of this population is thought to be seen with the expression of *wt1a* transcripts at the 1–4 somite stage (ss) [120]. The paralogue of *wt1a*, known as *wt1b*, is not expressed until after 10 ss and appears in a similar yet much more restricted spatial pattern that is believed to specifically demarcate the podocyte lineage [123,124].

In addition to *wt1b*, by the 15 ss the two small, spherical fields of podocytes are also visualized based on their expression of markers such as *hey1, lhx1a*, and *mafba* [124]. Studies have also shown that the transcription factor *osr1* is coexpressed in *wt1a*+ cells during this period, though it is also expressed in endoderm [148]. At the 24 hpf stage, the podocyte fields can be discerned by *wt1a, wt1b, lhx1a*, and *mafba* (Figure 5) [73,92,123,124]. Around this stage, transcripts encoding various slit diaphragm markers, such as *nphs1*, *nphs2*, and *podocalyxin*, also become visible [124]. After 30 hpf, podocytes begin to migrate towards the midline. By the 48 hpf stage, these bilateral groups of podocytes meet at the midline and form one conglomerate that expresses *vascular endothelial growth factor* (*vegf*) and *angiopoietin2* [92,149,150,151,152]. Secretion of these factors promotes angiogenesis and more specifically works to attract endothelial cells that express the VEGF receptor *flk1* to the site (Figure 5) [92,149,150,151,152]. The zebrafish model has been a very useful platform to observe podocyte migration and differentiation [149,150,151,152,153,154,155,156]. For example, *magi2a* marks podocytes with a similar pattern to *nphs2* at this time point, though further studies are needed to elucidate the full expression time course [152].

While not all antibodies used in mammalian studies are compatible with zebrafish samples, there has been progress in establishing reagents and protocols for visualizing zebrafish podocyte-associated proteins through immunohistochemistry in sections and whole-mount. For example, the slit-diaphragm has been visualized between 2–5 days post fertilization (dpf) using Nphs1, Nphs2, and podocalyxin antibodies [153,154,155,156].

### 3.2. Zebrafish In Vivo Podocyte Functional Assays

A major benefit of using zebrafish for research is that the living zebrafish embryo can be non-invasively imaged as it grows due to its ex vivo development. For studies of nephron function, this allows researchers to assess the ability of the renal corpuscle to filter blood in real time. There are several transgenic zebrafish lines that make these experiments achievable. There are *wt1a* and *wt1b* reporter lines that have been utilized in multiple studies [123,157,158,159,160,161]. There are also lines such as *nphs1*::GFP and *nphs2*::GFP that demarcate the slit diaphragm. Combining these lines with vasculature transgenics such as *flk1*::GFP allows for the co-visualization of the glomerulus and podocytes.

Of note, inducible podocyte ablation lines have been generated and used to model injury [162,163]. For example, the line *pod::NTR-mCherry* is driven under the *nphs2* promoter (formerly known as *podocin*) and marks the slit diaphragm with red fluorescent protein. These podocytes also express bacterial nitroreductase (NTR), which reduces the normally benign chemical metronidazole (MTZ), producing a cytotoxic effect [162,163]. This inducible podocyte-damage line has also been used to follow podocyte movements after administered injury, where researchers found that damaged cells are largely static in a 24-h post-injury period [160].

The functionality of the renal corpuscle can be assessed by the injection of fluorescent dextran dyes into the vasculature and monitoring rates of passage in the nephron tubule [164,165,166]. Additionally, transgenics have been created that enable researchers to measure the integrity of the glomerulus [162]. As vitamin-D binding protein (VDBP) is unable to pass the blood-brain barrier or analogous glomerular filtration barrier, the absence of VDBP in circulation and its accumulation in the pronephric tubules are used as an indication that glomerular filtration has failed [162]. There are wide-ranging applications for this transgenic line that have already been utilized across various studies [164,165,166,167].

## 4. Identification of Zebrafish Podocyte Developmental Pathways

### 4.1. Transcription Factors and Signaling Pathways

In order to improve therapeutics, it is essential to improve our working knowledge of how a podocyte is made. One of the first requirements to make a zebrafish podocyte is the correct expression of the transcription factors *wt1a/b*. As stated previously, *wt1a* is one of the earliest genes to be expressed in zebrafish that is associated with podocytes, but it is not specific to podocyte cells alone. It is also thought to give rise to the interrenal gland populace, the fish equivalent of the adrenal gland, and is expressed in other organs such as the epicardium [120,136]. Decreases in podocyte populaces are often associated with increases in interrenal gland cells due to cell fate changes, possibly in the *wt1a+* cells [124,168,169]. Of the *wt1* paralogs, *wt1a* is thought to be the dominant of the two genes as morpholino-induced knockdown of *wt1a* leads to a dramatic loss in slit diaphragm markers *nphs1* and *nphs2* while *wt1b* morpholino knockdown only leads to a partial reduction in these markers [122,123,124]. In contrast, another study has shown that knockdown of *wt1a* produces a similar loss in *nphs2, mafba*, and *magi2a* as knockdown of *wt1b* alone [152]. While the exact timing and dosage of these genes is still unknown, it is clear that both of the *wt1* genes are required for podocyte formation, as their loss supersedes the effects of other genetic influences [104].

Another early podocyte program prerequisite is early exposure to retinoic acid (RA) [170]. RA is a morphogen that is secreted by neighboring paraxial mesodermal cells slated to become somites [93,94]. Exposure to exogenous RA causes an increase in podocytes and proximal tubule domains, while inhibition of RA with the chemical DEAB causes a diminishment in these cells [93,94,168,169,171]. Further, it has been shown in zebrafish that the promoter of *wt1a* contains RA response elements (RAREs) and responds to RA receptor signaling [172].

After exposure to RA, podocytes are further promoted by Notch signaling. The podocyte field expands in mutant progeny of the Notch intracellular domain (NICD) heat-shock transgenic line and decreases due to Notch inhibition through gamma-secretase inhibitors [124,169]. Interestingly, O’Brien et al. found that Notch signaling components NICD and Maml form a complex with Wt1 and FoxC1/2 to promote podocyte formation through the transcription of *hey1* [124].

Similar to both the human and mouse, the transcription factor *dachd* was found to be localized to podocytes in zebrafish [159]. Morpholino-induced knockdown of *dachd* led to a reduction in glomerular filtration and podocyte effacement, as seen by *wt1a::GFP* and *nphs1*. Intriguingly, the mammalian counterpart, DACH1, was also reduced in effaced SYNPO-expressing podocytes in diabetic nephropathy patients [159].

### 4.2. Development of the Slit Diaphragm

Each slit diaphragm protein is integral to maintaining the selectivity of the blood filter. Two of the most widely studied of these proteins are Nphs1 and Nphs2. Knockdown of *nphs1* and *nphs2* using morpholinos causes disruption of the slit diaphragm and glomerular filtration [173]. In one study, it was shown that while wild-type mRNA could rescue *npsh1* and *nphs2* morphants, mRNA that contained human-disease causing mutations in *nphs1* and *nphs2* delivered a less effective rescue [173]. Both of these genes are regulated by several transcription factors. For example, *nphs2* has been shown to be co-promoted by the transcription factors Lmx1b and Foxc1a [174].

Similarly, knockdown of *podocalyxin* using morpholinos resulted in the incomplete development of secondary foot processes and thus reduced slit diaphragm integrity [126]. Yet another factor that is essential to slit diaphragm integrity in both zebrafish and mammals is *cd2-associated protein* (*cd2ap*), and animals lacking this protein exhibit podocyte effacement, edema, and the symptoms of the nephrotic syndrome [165,175,176]. Protein-protein interactions between slit diaphragm components Neph1 and ZO-1 are also crucial for integrity [177]. Collectively, these genes are needed to ensure proper foot process architecture as well as establish and/or maintain the mature slit diaphragm.

### 4.3. Identification of Novel Podocyte Developmental Factors through Zebrafish Screens

In addition to the rapid organogenesis of the zebrafish embryo, another major advantage of the model is the fecundity of the adults, who produce large egg clutch sizes of 100–1000 embryos or more on a weekly basis. This feature makes the zebrafish model amenable to high-throughput screening. These screens have been performed to find previously unknown genetic regulators of podocyte development. Several methodologies have been employed, such as reverse candidate screens, forward mutagenesis screens, and chemical screens [76,92,124,153,169,178,179,180,181].

One example of a candidate screen was performed by Ebarasi et al. [153]. Previously, GlomBase was compiled to report genes that were enriched in mouse glomeruli [182]. To determine if any of these genes were functionally critical to podocyte development, Ebarasi et al. [153] used morpholinos to perform knockdowns of GlomBase genes. They then used a glomerular filtration assay to determine changes in filtration functionality in live, 3.5–4-day-old embryos. They found that knocking down the gene *crb2b* led to simplification of foot processes and a significant reduction in the filtration ability of the slit diaphragm. Additionally, *nphs1* is mislocalized in the morphant embryos, further suggesting that *crb2b* is required for the formation of the slit diaphragm. A follow-up study found that knockdown of *crb2b* in podocin-GFP lines also resulted in a loss of normal slit-diaphragm structures [179]. This study also implicated the genes *ralgps1, rapgef2*, and *rabgef1* as being important for correct glomerular filtration. An additional hit from GlomBase, *tmem234*, was worked up by Rodriguez et al. [183]. They found that morpholino-induced knockdown of *tmem234* led to edema and dysmorphic glomeruli. Disruption of the transmembrane protein Tmem63c is also crucial to podocyte physiology, where knockdown leads to podocyte effacement associated with ultrastructural defects in the slit diaphragm [184].

Random mutagenesis screens are forward-looking genetic approaches that entail creating mutant lines based on phenotypes of interest and then determining what genetic lesions are responsible for them. One method utilizes the mutagen ethylnitrosourea (ENU) to introduce point mutations. With this mutagen, an F3 screen was performed to find recessive alleles that were associated with the development of edema, which is a hallmark of renal malfunction [169]. One mutant from this screen was named *zeppelin* (*zep*) on the basis of its distinctive blimp-like edema at the 96 hpf stage, which we found was due to the complete absence of podocytes and glomerulus formation in the mutants [169]. Whole genome sequencing revealed that the causative mutation was in *breast cancer 2, early onset (brca2)/fancd1*, a gene well known for its causative contributions to various cancers and genetic diseases such as Fanconi anemia (FA) [169]. While previous research linked human BRCA2 mutations to renal anomalies such as the horseshoe kidney and pediatric renal cancer, a role for this gene in vertebrate podocyte development was previously unknown [185,186,187,188,189,190,191,192,193,194]. Interestingly, while zebrafish embryonic podocyte emergence is reliant on *brca2*, a concomitant increase in the interrenal lineage occurs in the mutants, possibly suggesting a role for Brca2 in intermediate mesoderm fate specification [169].

Another mutant, *oceanside* (*ocn*), was discovered by our lab in a haploid forward ENU screen in which whole mount in situ hybridization was performed to directly query the formation of podocytes, proximal tubule, and distal early tubule lineages [115,179]. *ocn* was distinctive in that mutants displayed an absence of podocytes at the 24 hpf stage, followed by edema that began by 48 hpf [195]. The genetic lesion in *ocn* is a mutation in the transcription factor *odd skipped-related 1* (*osr1*) and was similarly identified using whole genome sequencing [195,196,197]. Previous studies have demonstrated key roles for *osr1* in regulating zebrafish pronephros development by influencing lineage specification [198,199,200,201,202,203]. In further characterizing podocyte development in *ocn*, we found that the expression of several markers was reduced in the rostral region of the early pronephros, among them *wnt2ba*. Previous findings had suggested co-expression of *wnt2ba* and *osr1* in the pronephros anlage [199], which we observed as well using double fluorescent in situ hybridization and confocal imaging, and led us to explore whether there was a relationship between *wnt2ba* and podocyte formation [195]. However, the *wnt2ba* loss of function was associated with a significant decrease in podocyte number, thereby implicating *wnt2ba* for the first time as necessary for the proper development of podocytes in the zebrafish embryo [195].

### 4.4. Zebrafish Models of Common and Rare Podocytopathies

The term “podocytopathy” is used to refer to diseases that are believed to initiate with podocyte damage or dysfunction [204,205,206]. Given the conservation of human and zebrafish podocytes, zebrafish have been used to model a number of podocytopathies. Nephrotic syndromes are a subclass of CKD that are known for high proteinuria, hyperlipidemia, hypoalbuminemia, and edema due to loss of the slit diaphragm [207]. Nephrotic syndromes can be further classified by sensitivity to steroids (SSNS) and resistance to steroid-based treatments (SRNS). SRNS often progresses to end-stage renal disease in patients. To identify genetic causes of SRNS, several studies have been conducted that performed whole genome/exome sequencing on patients with this condition. One monogenic cause of SRNS that was identified in this way was EMP2, which was further examined in zebrafish models [207,208]. Multiple zebrafish loss of function models demonstrated that knockdown of *emp2* resulted in edema as well as the upregulation of *cav1* [207,208]. Additionally, glucocorticoid treatment lessened the detrimental effects of this *cav1* overexpression [208]. CRB2 mutations were also identified in families affected by SRNS, and a *crb2b* zebrafish mutant line was created that expanded on previous findings in *crb2b* morphants that this gene is critical for apical-basal polarity in podocytes [153,179]. Furthermore, FAT1 mutations are linked to SRNS, and Fat1 knock down in mice and zebrafish resulted in similar podocyte effacement and tubular cysts [209].

Zebrafish have been used by many labs to test the function of genes associated with focal segmental glomerulosclerosis (FSGS). In FSGS, scar formation occurs within the glomerulus, and podocytes exhibit effacement, injury, and death. Upon identification of human mutations linked to FSGS, the zebrafish offers a convenient opportunity to assess whether disruption of a candidate gene alters podocyte structure and function. Studies of candidates such as ANLN, for example, have used zebrafish embryos to test physiological processes such as filtration, dynamics of glomerular filtration barrier integrity and then examine the foot process ultrastructure [210,211]. Similar strategies have been used to test other genes as well [212]. Zebrafish have also been used to test the circulating permeability factors in patient serum [213] and to examine whether administration of materials such as growth factors might ameliorate podocyte damage [214].

The features of the zebrafish model also allow for the study of rare and neglected kidney diseases. APOL1-associated nephropathy has an increased prevalence in patients of African ancestry and, when combined with additional risk factors, can lead to ESRD [215,216]. While co-expressed with NPHS1 in humans and zebrafish, interestingly, this expression pattern is not conserved in mice [216]. Morpholino-induced knockdown of *apol1* in zebrafish embryos led to malformations in the slit diaphragm, which were salvageable with human NPHS1 mRNA. As another example, Elmonem et al. generated *ctns* mutant zebrafish to model nephropathic cystinosis [217].

### 4.5. Zebrafish as a Model of Podocyte Regeneration

It has been known for decades that some fish are capable of robust renal growth and regeneration throughout their lives. Two phenomena have been observed: (1) replacement of epithelial populations within existing nephrons; and (2) the formation of new nephrons, also known as neonephrogenesis, during adult growth or in response to catastrophic organ damage [218]. The latter phenomenon was first observed in goldfish that had been treated with the nephrotoxin hexachlorobutadiene and formed new nephrons after several weeks [219,220,221,222,223,224]. This was observed by an increase in DNA replication, as indicated by the incorporation of the nucleotide analog bromodeoxyuridine, followed by an increase in the percentage kidney volume in of injured goldfish [219,220,221,222,223,224]. By comparison, in mammals, if the basement membrane of the nephron remains intact, renal regeneration occurs by just the first phenomenon listed above, through the formation of a wave of flattened mesenchymal cells that differentiate into the required specialized epithelia [218]. Thus, mammals only exhibit nephron genesis during gestation and sometimes during early post-natal stages, the timing and scale of which are traits that vary across species, and never form new nephrons during adulthood [225,226,227]. However, both of these forms of renal regeneration have now been reported in a variety of other fish, including the zebrafish, catfish, trout, medaka, and tilapia [228,229,230,231,232].

Amongst these possible animal models, the advancement of sophisticated genetic tools and methodologies in the zebrafish offers a particularly appealing avenue to delineate the mechanisms of renal regeneration events in various lineages, such as the podocytes [73,74,75,119,157,233,234,235,236,237,238]. As previously mentioned, several inducible podocyte injury models have been created that enable the specific abrogation of this lineage, and then the subsequent experimental analysis of the underlying events [162,163]. Furthermore, a number of studies have provided the initial molecular framework to track and decipher such phenomena using chemical injury models in the adult kidney to damage nephrons and thereby stimulate neonephrogenesis from resident renal progenitors. For example, in zebrafish juveniles and adults, new nephrons first appear as clusters of renal progenitors near existing mesonephros tubules [119,157]. These clusters express early markers of renal development such as *lhx1a, pax2a, wt1b*, and *pax8*, which suggests that developmental pathways mirror pathways of regeneration [119,157,238]. These clusters then expand into S-shaped bodies that mature into nephrons that fuse with preexisting nephrons [119,157,238]. Understanding the molecular attributes of these cells is an attractive option to identify cell features that could allow for similar events to be induced in humans, and there has been ongoing progress in identifying signaling pathways and cell interactions that mediate nephron regeneration in zebrafish [239,240,241,242].

## 5. Discussion

Ongoing research about podocyte development and regeneration offers many opportunities to better understand and treat renal conditions. While our focus in the present work has been to highlight contemporary advances courtesy of work in the zebrafish model, research in mammalian models has yielded many important advances as well. For example, juvenile rodents exhibit some capacity for de novo podocyte production from glomerular parietal epithelial cells [243], and recent studies in rodents now suggest that podocytes may regenerate in the adult kidney [244,245,246,247]. While these observations are still under active investigation, they raise many intriguing questions about whether these properties exist in humans. In the discussion sections below, we explore several aspects for continued future study.

### 5.1. Applying Genetic Knowledge to Advance Organoid Technology

In recent years, many breakthroughs have been made in the field of kidney organoids [51,52,53,54,55,56,57,58,59,60,61,62,63]. These 3D cell cultures could potentially allow scientists to grow patient-specific specimens that could be used to assess drug toxicity and disease progression [62,63]. The dream, of course, would be to advance kidney organoids to the point of being useful for transplantation. However, the field is far from this goal. While many kidney cell types are present in these organoids, progress is still needed to induce fully differentiated, completely functional, and mature kidney cells [248]. Continued discovery of genetic players would give scientists new insights into the development of novel cultural conditions.

One approach to finding genes of interest is to employ nucleic acid sequencing, or “seq,” technology to analyze the genetic contents of a kidney cell. Within the last few years alone, a bevy of studies have been published that utilized single cell RNA-seq to profile the transcriptomes of various murine and human renal cell types over the course of development [249,250,251,252,253,254,255,256]. While these studies have shed light on the origins of renal cells and identified new genes that are associated with these cells, further research is needed to determine which genes are truly required for the continuation of kidney development. The only way to evaluate the potency of a gene on the development process is through loss and gain of function studies. Given the limitations of mammalian models, zebrafish studies are one feasible approach to such studies.

For example, two factors that were unappreciated in kidney development until our studies but could potentially promote podocyte formation in organoids are *wnt2ba* and *brca2*. Both of these genes were shown to be necessary and sufficient for podocyte maturation. In the future, studies should assess if these genes function along the same or alternate pathways and whether or not they interact directly or indirectly. It is hypothesized that *wnt2ba* functions downstream of *brca2* given that loss of *wnt2ba* leads to a decrease in podocyte area while *brca2* abrogates podocytes completely. It would be interesting to see if abrogation of *BRCA2* in kidney organoids similarly affects podocyte development. It would be equally fascinating to see if *BRCA2* and/or *WNT2B* could stimulate ectopic podocytes in culture, as they can in the zebrafish model.

### 5.2. Brca2 Mechanisms in Podocyte Development and Diseases

Similar to its mammalian counterpart, zebrafish *brca2* has previously been implicated in acting as a tumor suppressor through a complex it forms to repair double-stranded DNA breaks [257,258,259]. Deficiency of another critical member of this complex, *rad51*, has recently been studied in zebrafish and shown to display symptoms of FA and smaller kidneys [260]. In light of this, other gene members of the FA pathway should be more closely scrutinized in the context of podocyte and kidney development to assess whether diminished DNA repair by other FA genes is causative of the fate switch seen between podocytes and the interrenal lineage in *zep* mutants. Continued study of FA has accumulated a list of genes whose mutations result in the disease, and the current list of these is curated on the Fanconi Anemia Mutation Database (http://www.rockefeller.edu/fanconi/ URL accessed 1 January 2023), a publicly available resource. Another aspect to consider is the variation of interrenal and podocyte phenotypes seen in *zep* and *brca2*^ZM_00057434^ homozygous mutants, as this possibly suggests that only a portion of the Brca2 protein is critical in regulating podocyte development.

Furthermore, there is a clinical link between FA patients who have bi-allelic *BRCA2* mutations and kidney diseases and malformations that has been appreciated but never understood [187,188,189,190,191,191]. There have also been clinical links between *BRCA2* mutations and adrenal cortical carcinoma [261]. Our observations that *brca2* mutations cause expanded interrenal glands in a developmental context suggest that adrenal gland neoplasms and the prevalence of kidney disorders in Fanconi anemia patients could be caused by mutations in *BRCA2*. Recently, Sathyanarayana et al. published a case study that found 50% of examined FA patients had renal anomalies and gave an official recommendation that, upon diagnosis of FA, patient kidneys should be monitored through ultrasound and other parameters [193].

Future studies are needed to determine if *BRCA2* is capable of influencing renal progenitors during mammalian kidney development. In mammalian development, three successive kidney forms develop and degrade: the pronephros, the mesonephros, and the terminal kidney, the metanephros [22]. While the major cell components of the renal corpuscle and nephron are conserved between the mammalian and teleost systems, zebrafish only form a pronephros followed by a mesonephros. Related to this difference, it is possible that BRCA2 only plays a role in the earliest kidney forms in mammalian development and may be less effective in controlling podocyte fate than in zebrafish. In opposition to this notion, it is also possible that *brca2* is a more potent factor in regulating kidney development than we have reported, as we have not exhaustively characterized all the nephron cell types or each time point critical to kidney development in *zep* mutants.

Looking forward, given the strengths of the zebrafish system for studying glomerular development and its amenability to chemical genetics, our *brca2* model affords new opportunities to identify modulators of Brca2 that influence mesoderm formation and other tissues during ontogeny. Our findings indicate that *brca2* can be considered in a new light as a participant in aspects of both development and cancer that may have important ramifications and biomedical significance for humans as well.

### 5.3. Zebrafish for CAKUT and Kidney Agenesis Research

The majority of ESRD cases in children are due to CAKUT [45,46,47,48,49]. Significantly, while over 75 causative genes have been identified, this only explains 10–15% of cases [262]. The most severe of all CAKUT cases are those where the kidney fails to develop at all, known as either unilateral or bilateral kidney agenesis [45,46,47,48,49]. Embryos with bilateral kidney agenesis rarely survive without interventions such as amnioinfusion and transplants [263]. While unilateral renal agenesis (URA), also known as congenital solitary functioning kidney, can be non-symptomatic in patients, at least 50% of them have congenital malformations in other tissues [45,46,47,48,49]. Thus, improving our understanding of renal agenesis has the potential to advance our broader knowledge of development as well.

Given that adult zebrafish have one kidney rather than the two that mice and humans have, there are obvious challenges to modeling renal agenesis in this system. One study sought to establish what the loss of a known URA gene would look similar to zebrafish. Brophy et al. first conducted whole exome sequencing in two families with inherited unilateral renal agenesis patients and found recurring mutations in *GREB1L* [264]. They then examined the kidneys of zebrafish embryos with *greb1l* mutations and found that the pronephros was dilated and contained cysts. However, the overall pronephros structure appeared to be intact. They did not disclose if mutants were viable and, if so, what the look of the adult mutant structures would be.

Our recent study of *osr1* using the *ocn* mutant model, which possesses a premature stop codon in exon 2, displayed a truncated pronephros from the earliest stages of development [194]. While our mutant is not viable, this is consistent with studies that found human embryos with *OSR1* mutations similarly do not carry to term [265]. However, as human carriers of *OSR1* mutations can have hypomorphic kidneys, it would be useful to characterize adult kidneys in *ocn* heterozygote carriers [266,267]. Kidney volume, nephron endowment, and nephron composition could all potentially be altered in *ocn*+/− animals. If any of these conditions vary from their WT counterparts, this could become a contemporary model to study conditions such as kidney hypoplasia.

There are an equally limited number of papers where the pronephros structure is reduced. Other than loss of *osr1*, there is only one other known genetic aberration that causes a similar anterior truncation of the zebrafish pronephros: the Wnt pathway. Lyons et al. showed that inhibition of canonical Wnt/β-catenin pathway by overexpression of ligand *dkk1* in a heat-shock zebrafish transgenic line caused a stunted proximal tubule and dysfunctional PCT at 3 dpf [268]. In the future, it would be useful to test if *osr1* cRNA is sufficient to rescue overexpression of the ligand *dkk1* heat shock.

Knowledge about key regulators of nephrogenesis is useful in a number of ways. CAKUT is a global issue with few explanations and many opportunities for further study using zebrafish [269,270]. If the genetic causes of these congenital problems were elucidated, they could be screened during early pregnancy or during genetic counseling sessions and bring critical knowledge to the affected families. Identifying the genes that are active in promoting and inhibiting kidney cell differentiation could also be used to develop targeted therapeutics.

### 5.4. New Vistas Await: Visualizing Renal Morphogenesis and Physiology In Vivo Using Zebrafish

The architecture, location, and rapid development of the zebrafish embryonic kidney are traits that make this system particularly well suited to observe organ morphogenesis. With respect to the kidney, the zebrafish continues to be an outstanding system for visualizing glomerulus formation. For example, they have enabled living imaging of calcium signaling in developing podocytes [271], and made exciting observations of how blood flow regulates glomerular capillary formation [272]. Further, glomerular architecture in response to signaling has been examined, with new insights uncovered for the role of prostaglandin signaling [273]. Transgenics have also been leveraged so as to observe normal filtration with subsequent proximal tubule reabsorption and compare these events to cases of proteinuria and altered cargo processing [274]. The development of various labeled tracers has furthered the assessment of passive glomerular filtration and other pharmacokinetic experiments [275].

## 6. Conclusions

In order for an organ to form correctly, precise actions need to be orchestrated by an elaborate cast of proteins and other signaling molecules in order to coordinate differential gene expression in an exact sequence. While genetic redundancy can buffer the effects of a misstep, individual genes can be so critical that development cannot continue as planned without them. In addition, the kidney is an organ that is vital to life, and dysfunction of this organ necessitates the formulation of the best medical interventions possible. Yet, the complexity of the mammalian kidney presents a number of difficulties for experimental studies. In contrast, the accessible, simplified zebrafish pronephros is a genetically tractable model to study kidney development. Podocytes are highly conserved between the zebrafish pronephros and mammalian metanephros. This has provided a valuable opportunity for experimental studies to delineate fundamental mechanisms of podocyte ontogeny, and the ongoing formulation of new methods and tools will continue to proffer innovative ways to tackle the plethora of questions that remain. As such, continued research utilizing the zebrafish will be a powerful tool for the nephrology community in the years to come as a means to advance aspects of biomedical research ranging from renal organoid technology to new therapeutics.

## Data Availability

Not applicable.
